# Smear layer removal by EDTA, citric acid, and maleic acid under five irrigant delivery and activation protocols: an *ex vivo* scanning electron microscopy study

**DOI:** 10.3389/fdmed.2026.1982158

**Published:** 2026-10-05

**Authors:** E. Hernández-Hernández, J. Mena-Alvarez

**Affiliations:** 1 Faculty of Dentistry, Alfonso X El Sabio University, Madrid, Spain; 2 Departments of Conservative Dentistry and Prosthetics, Faculty of Dentistry, Complutense University of Madrid, Madrid, Spain

**Keywords:** chelating agents, citric acid, EDTA, endodontic irrigation, maleic acid, passive ultrasonic irrigation, scanning electron microscopy, smear layer

## Abstract

**Background/purpose:**

This randomized *ex vivo* study evaluated the effects of chelator, irrigant delivery/activation, and root third on smear-layer removal and whether conclusions depend on the scanning electron microscopy (SEM) assessment method.

**Material and methods:**

Of 215 extracted single-rooted teeth, five served as descriptive controls and 210 were randomized to a 3 × 5 factorial design (*n* = 14/cell): 17% EDTA, 15% citric acid, or 7% maleic acid combined with syringe irrigation, manual dynamic activation, Endo Activator, passive ultrasonic irrigation (PUI), or EndoVac. Coronal, middle, and apical SEM fields were assessed using the Prabhu score, Carvalho clean-tubule percentage, and tubule diameter. Cluster-aware generalized estimating equations modeled the three repeated root-level observations using the tooth as the clustering unit; *post hoc* contrasts used Holm adjustment.

**Results:**

For Prabhu scores, chelator x delivery/activation, chelator x root-third, and delivery/activation x root-third interactions were significant (all *p* < 0.001). For clean-tubule percentage, chelator x root-third (*p* = 0.040) and delivery/activation x root-third (*p* = 0.020) were significant, whereas chelator x delivery/activation was not (*p* = 0.090). In the apical third, model-estimated marginal Prabhu scores were 2.80 for 17% EDTA, 0.93 for 15% citric acid, and 1.85 for 7% maleic acid; corresponding model-estimated clean-tubule percentages were 26.1%, 72.7%, and 54.4%. PUI had the lowest Prabhu estimates, but no apical quantitative pairwise delivery/activation contrast remained significant after Holm adjustment. The two SEM assessment approaches showed a moderate inverse association (Spearman *ρ* = −0.486); a threshold-dependent weighted *κ* analysis was retained only as a sensitivity analysis.

**Conclusions:**

Chelator selection consistently influenced smear-layer removal, whereas delivery/activation effects depended on root level and assessment method. Under the present experimental conditions, 15% citric acid showed the most favorable apical SEM performance. These findings concern laboratory surface-cleanliness endpoints and do not establish clinical superiority or an optimal chelating protocol.

## 1 Introduction

Successful root canal treatment depends on reducing the microbial burden and removing organic and inorganic remnants from a complex canal system. Mechanical preparation is indispensable, but it leaves areas untouched and generates a smear layer composed of dentin debris, organic tissue, microorganisms, and odontoblastic process remnants (1–4). The smear layer may reduce irrigants penetration, limit intracanal-medication activity, and interfere with the adaptation and penetration of obturation materials. Its complete removal, however, has not been established as a direct surrogate for long-term clinical success; laboratory cleanliness outcomes must therefore be interpreted as mechanistic rather than patient-centered endpoints (2–5).

Sodium hypochlorite remains the principal irrigant for organic tissue dissolution and antimicrobial action, but it does not predictably remove the inorganic component of the smear layer. Chelating or acidic solutions are consequently used during final irrigation. EDTA is the most established agent, while citric acid and maleic acid have been investigated as alternatives with different demineralizing kinetics, surface effects, and activity in the apical third (5–10). Recent evidence syntheses identify clinically relevant trade-offs between smear removal and dentin erosion, microhardness reduction, or alteration of the mineral matrix (5–7, 11, 12). Contemporary irrigation concepts therefore extend beyond disinfection to conditioning of the dentin substrate (13). An ideal protocol would provide adequate smear removal while limiting undesirable substrate alteration; the present study can evaluate the former but cannot directly determine the latter because dentin erosion and mineral loss were not measured.

Chemical effectiveness also depends on irrigant exchange and hydrodynamics. Conventional syringe irrigation may be limited by needle penetration, stagnant fluid, and apical vapor lock. Manual dynamic activation, sonic agitation, PUI, and apical negative pressure have been proposed to enhance fluid replacement and canal-wall contact (2, 3, 13–18). These approaches are not mechanistically equivalent: EndoVac is primarily a delivery/aspiration system, whereas sonic and ultrasonic devices agitate the solution. Results remain heterogeneous because studies differ in root anatomy, preparation size, irrigant sequence, activation time, and the method used to evaluate SEM images (17–21).

Most smear-layer SEM studies use ordinal visual scores. Such scores are practical but observer-dependent and may lose information by assigning a broad category to a complex microscopic field. Quantitative counting of clean and occluded tubules may improve reproducibility, although it samples only part of each image and is also sensitive to field selection (9, 19, 22). Direct comparison of qualitative and quantitative assessment within the same large factorial experiment is uncommon.

The primary objective of this study was to compare the smear-layer removal effectiveness of 17% EDTA, 15% citric acid, and 7% maleic acid when used with five irrigant delivery or activation protocols in the coronal, middle, and apical thirds of single-rooted human teeth. Secondary objectives were to quantify the association between qualitative and quantitative SEM assessment and to explore changes in mean dentinal tubule diameter. The primary null hypotheses were that chelator, delivery/activation protocol, and root third would not affect the measured outcomes and that no two-way interactions would occur among these factors. A full three-way interaction was evaluated as an exploratory sensitivity analysis.

## 2 Materials and methods

### 2.1 Study design, reporting framework, and ethics

This randomized *ex vivo* laboratory study used extracted human teeth and a 3 × 5 factorial experimental structure. The manuscript was prepared with reference to the PRILE 2021 reporting recommendations for laboratory studies in Endodontics (23). The study was approved by the Bioethics Committee of the Universidad Alfonso X el Sabio (reference number 2023_12/238). Written informed consent for use of the extracted teeth in research was obtained before extraction. All specimens were anonymized before laboratory procedures.

### 2.2 Specimen selection, sample size, and randomization

The sample comprised 215 extracted human single-rooted teeth stored in physiologic saline. Teeth were included when a single root and a single canal were confirmed clinically after decoronation with a DG16 explorer. Exclusion criteria were root caries, previous endodontic treatment, canal calcification, or absence of informed consent. The archived study records do not provide a tooth-type distribution, standardized root-curvature measurements, canal cross-sectional geometry, dentin thickness, or preoperative two-projection radiographic/CBCT/micro-CT characterization; therefore, equivalence of these anatomical features across the 15 experimental cells cannot be verified retrospectively. Five teeth were assigned to descriptive control conditions and were not included in inferential analyses. The remaining 210 teeth were assigned to 15 experimental groups. A previous pilot study informed an *a priori* GRANMO 7.12 sample-size calculation, which indicated 12–14 specimens per group; 14 specimens were used in every factorial cell. Random allocation was generated with EPIDAT 4.2 program (Dirección Xéral de Saude Pública, Galicia, Spain) (Figure 1).

**Figure 1:**
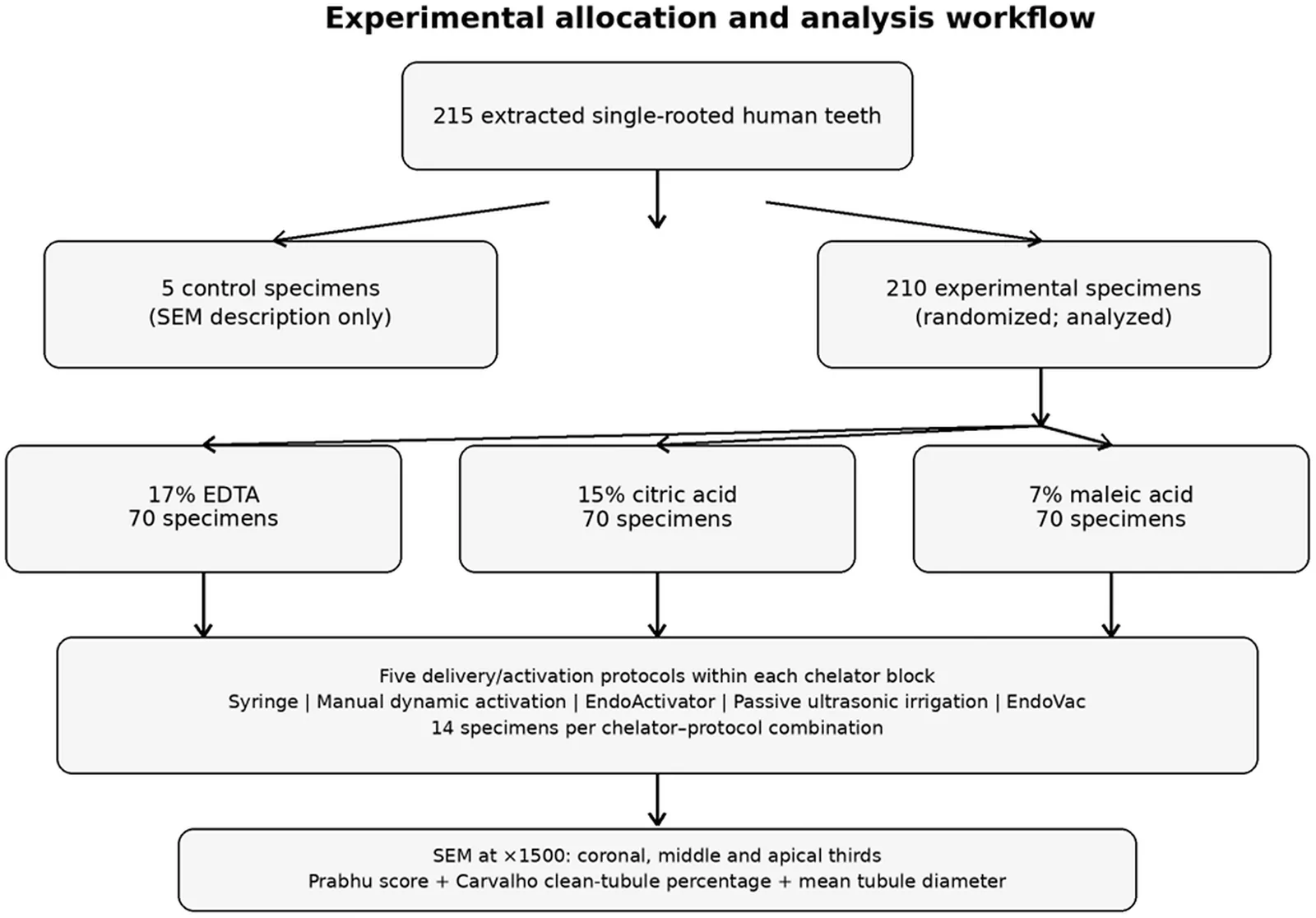
Experimental allocation and analysis workflow. Five control specimens were examined descriptively; 210 teeth were randomized across 15 chelator–delivery/activation combinations (*n* = 14 each).

### 2.3 Root canal preparation

Root surfaces were cleaned and crowns were removed to standardize root length at 14 mm. Working length was established by advancing a size 10 K-file until its tip was visible at the apical foramen and subtracting 1 mm. A manual glide path was prepared with sizes 10, 15, and 20 K-files. Canals were then shaped with ProTaper Gold ® (Dentsply-Sirona, Ballaigues, Switzerland) instruments in the sequence S1, SX, S2, F1, F2, and F3, followed by apical enlargement to ISO size 35 with a hand K-file. An apical enlargement to ISO size 35 was selected, as this was the minimum apical size required to use the EndoVac microcannula. No separate apical enlargement was performed beyond the procedure established for the working length. Apical patency was maintained with a size 10 K-file between instruments. During shaping, 2 mL of 5.25% sodium hypochlorite was delivered after each instrument through a 30-gauge Monoject needle positioned 2 mm short of working length. A cumulative shaping-irrigant volume was not recorded as a separate variable and has therefore not been reconstructed retrospectively.

To reproduce a closed-ended canal model, an Protaper F3® gutta-percha cone (Dentsply-Sirona, Ballaigues, Switzerland) was modified from its nominal ISO 30 tip to ISO 35 using a calibrated gutta-percha gauge, inserted to obtain an apical fit, and the external root surface was coated with molten wax. The model relied on the snug apical cone fit and wax coating to prevent apical fluid escape; no independent dye- or pressure-leakage test was recorded. The apical wax was maintained during final irrigation. This model was intended to reproduce a closed-end hydrodynamic condition.

### 2.4 Experimental groups and final irrigation

The 210 experimental teeth were divided into three chelator blocks: 17% EDTA, 15% citric acid, and 7% maleic acid (70 teeth each). Within every block, teeth were randomized to syringe irrigation, manual dynamic activation, Endo Activator® sonic activation (Dentsply-Sirona, Ballaigues, Switzerland), PUI (Suprasson P5 Booster® with Irri-Safe® (Satelec Acteon, Merignac, France), or EndoVac® (Kerr Endodontics, Gilbert AZ, USA) apical negative pressure delivery/aspiration with14 teeth per combination. The control teeth received sodium hypochlorite activated or delivered with one of the five systems but no chelating solution (Table 1). The tested chelator concentrations were established protocol-specific formulations and should not be interpreted as chemically or molarly equivalent exposures; accordingly, this study compares complete irrigation protocols rather than intrinsic molecule-for-molecule chelating potency.

**Table 1:** Experimental allocation (*n* = 14 per experimental cell).

Chelator	Syringe	Manual	EndoActivator	PUI	EndoVac	Total
17% EDTA	14	14	14	14	14	70
15% citric acid	14	14	14	14	14	70
7% maleic acid	14	14	14	14	14	70
Total	42	42	42	42	42	210

The final irrigation sequence was standardized across groups: 2 mL of 5.25% sodium hypochlorite delivered over 1 min and subjected to the assigned 20-s delivery/activation phase; 2 mL of 0.9% sodium chloride over 1 min without activation; 2 mL of the assigned chelating solution over 1 min with the assigned 20-s delivery/ activation phase; 2 mL of 0.9% sodium chloride over 1 min; and a final 2 mL of 5.25% sodium hypochlorite over 1 min with the assigned 20-s delivery/activation phase (Table 2). Because NaOCl was applied after the chelator, subsequent proteolytic exposure could also contribute to the final dentin-surface appearance; therefore, visible tubule diameter cannot be attributed to the chelating solution alone.

**Table 2:** Delivery and activation procedures used during the active 20-s phase.

Protocol	Operational description
Syringe irrigation	30-gauge needle positioned 2 mm short of working length; no adjunctive activation.
Manual dynamic activation	F3 gutta-percha cone adapted to ISO 35, positioned 2 mm short of working length; approximately 100 push–pull strokes/min for 20 s.
EndoActivator	Size 25/0.04 polymer tip at 10,000 cycles/min, 2 mm short of working length, with 2–3 mm vertical movements for 20 s. The tip was nominally smaller than the ISO 35 apical preparation; no separate rationale was documented in the archived protocol.
Passive ultrasonic irrigation	Suprasson P5 Booster with Irri-Safe 20/21 mm insert, device power setting 5, positioned 1 mm short of working length for 20 s. The protocol does not retain a manufacturer-equivalent physical power output or a systematic record of file-wall contact during activation.
EndoVac	Apical negative-pressure delivery/aspiration: macrocannula irrigation followed by microcannula placement at working length, 2 mm short of working length, and again at working length until 2 mL of each solution had been aspirated.

### 2.5 Specimen processing and scanning electron microscopy

A longitudinal external groove was prepared under water cooling with a high-speed diamond bur without entering the canal lumen. Root separation was completed with a scalpel to minimize creation of a new smear layer. Specimens were dehydrated in ascending ethanol concentrations (50%, 70%, 80%, 90%, 95%, and three changes of 100%; 1 h per step), critical-point dried (POLARON CPD7501), mounted, and sputter-coated with gold (BALZERS SCD004). Root canal walls were examined with a HITACHI S3000N scanning electron microscope at ×1,500. One photomicrograph was obtained from each coronal, middle, and apical third, generating three observations per tooth and more than 600 experimental images. The study identifies the root third but does not a coordinate-based or computer-randomized rule for selecting the exact SEM field within each third. Nor does it record systematic acquisition of multiple fields. Consequently, field-selection bias cannot be excluded and is addressed as a study limitation.

### 2.6 Outcome assessment

For the Prabhu method, each image was independently scored by two observers on the original 0–3 scale: 0, no smear layer and all tubules open; 1, slight smear layer with more than 50% of tubules open; 2, moderate smear layer with fewer than 50% of tubules open; and 3, heavy smear layer with all tubules occluded (9). Both observers were non-dentists, were unaware of the study objective and group allocation, and evaluated images on the same computer under standardized viewing distance, illumination, and image size. The arithmetic mean of the two observer scores was retained as the finalized image-level Prabhu value, so half-point values could occur. The separate observer-level ratings were not retained in the analysis dataset; consequently, inter-rater reliability cannot be recalculated.

For the quantitative Carvalho approach, digital images were displayed at 29 × 22 cm and a 10 × 8 cm template window was placed at a randomly selected position on each image, following the Carvalho method with the sampling concept described by Mayer et al. (19, 20). Within the window, patent/open tubules free of visible occluding smear were counted as clean dentinal tubules (CDT), whereas visibly occluded tubules were counted as dirty dentinal tubules (DDT); the percentage of clean tubules was calculated as CDT/(CDT + DDT) × 100 (19, 22). The following descriptive categories were used: 81%–100%, very clean; 61%–80%, clean; 41%–60%, moderate; 21%–40%, dirty; and 0%–20%, very dirty. Quantitative analysis was performed by a blinded observer. The continuous percentage, rather than the descriptive category, was used for the primary quantitative inferential analysis.

Mean dentinal tubule diameter was measured in the same sampling with Adobe Photoshop CS3® (Adobe Systems, San Jose, CA, USA). Calibration was performed from the micrometric scale printed on each SEM image: distances measured on screen were converted to µm by proportional scaling to the image scale. The mesiodistal diameter of every visible tubule within the window was measured and averaged to obtain one mean value per image. Because the number and identity of measurable tubules necessarily depend on how much of the lumen remains visible, heavily smeared fields may preferentially contribute the most patent tubules. Tubule diameter was therefore considered an exploratory morphologic outcome and not a direct measure of either cleaning efficacy or dentin erosion.

### 2.7 Statistical analysis

Descriptive statistics included means, medians, standard deviations, and 95% confidence intervals. Because each experimental tooth contributed three SEM observations (coronal, middle, and apical), all primary inferential analyses explicitly modeled within-tooth correlation using tooth as the clustering unit and an exchangeable working correlation structure. The finalized Prabhu value (the mean of two original 0–3 observer scores) was treated as an ordered response in a cumulative-logit GEE. The proportional-odds structure was selected to preserve the ordered nature of the endpoint while limiting model complexity; a formal proportional-odds diagnostic was not performed in the GEE reanalysis and this is acknowledged as a modeling limitation. Model-estimated Prabhu values reported in the Results are expected scores derived from the model-predicted probabilities across the ordered score levels, rather than directly fitted continuous means. The clean-tubule percentage was scaled to the closed 0–1 interval and analyzed with a fractional-logit GEE using Bernoulli quasi-likelihood; boundary proportions, if present, were retained without transformation because the fractional-logit mean model accommodates values at 0 and 1. Mean dentinal tubule diameter was analyzed with a Gaussian identity-link GEE. Wald tests labeled as joint factor-block tests evaluated the main-effect coefficients together with every two-way interaction involving that factor; consequently, their degrees of freedom exceed the simple factor-level df (chelator: 2 + 8 + 4 = 14; delivery/activation: 4 + 8 + 8 = 20; root third: 2 + 4 + 8 = 14). Conventional interaction-block tests are reported separately. A full chelator  ×  delivery/activation×root-third interaction was fitted only as an exploratory sensitivity analysis. Because this model estimates many parameters from 14 teeth per factorial cell, no exhaustive cell-level *post hoc* decomposition of the three-way interaction was undertaken. Model-estimated marginal means with 95% confidence intervals were calculated by chelator and root third and by delivery/activation protocol and root third; pairwise contrasts were adjusted with the Holm procedure within each outcome-by-root-third comparison family. The primary comparison between SEM assessment approaches was Spearman correlation between finalized Prabhu value and continuous clean-tubule percentage, with a confidence interval obtained by bootstrap resampling at tooth level. Weighted Cohen *κ* was retained only as a secondary sensitivity analysis after pragmatic recategorization of the quantitative percentages (81%–100% = 0; 61%–80% = 1; 41%–60% = 2; 0%–40% = 3). Because these thresholds are not naturally equivalent to the Prabhu definitions, *κ* was not interpreted as a criterion-validity measure. All tests were two-sided with alpha=0.05. The cluster-aware reanalysis was performed in Python 3.13 using statsmodels 0.14.6 and SciPy 1.17.0.

## 3 Results

### 3.1 Specimen flow and data integrity

All 215 teeth entered the planned laboratory workflow. Five controls were examined descriptively and 210 experimental specimens contributed to the inferential analysis. Each experimental tooth yielded one coronal, one middle, and one apical SEM field, producing 630 clustered observations. All 15 chelator×delivery/activation cells contained 14 teeth, and no post-randomization experimental exclusions were identified. The dataset retained finalized image-level Prabhu values, clean-tubule percentages, and mean tubule diameters, but not the separate observer ratings or the original CDT/DDT counts; therefore, observer-specific score distributions and binomial count tables could not be reconstructed from the archived records.

### 3.2 Factorial cluster-aware analysis

The effects of the experimental factors depended on the other factors, as demonstrated by the interaction terms. For the Prabhu outcome, all three two-way interactions were significant: chelator×delivery/activation (Wald *χ*^2^ = 42.19, df = 8, *p* < 0.001), chelator×root third (*χ*^2^ = 29.96, df = 4, *p* < 0.001), and delivery/activation×root third (*χ*^2^ = 59.25, df = 8, *p* < 0.001). For the quantitative clean-tubule percentage, chelator×root third (*χ*^2^ = 10.03, df = 4, *p* = 0.040) and delivry/activation×root third (*χ*^2^ = 18.16, df = 8, *p* = 0.020) were significant, whereas chelator×delivery/activation was not (*χ*^2^ = 13.69, df = 8, *p* = 0.090). The joint factor-block tests in Table 3 include the main effect and all two-way interaction terms involving each factor and are not simple main-effect tests.

**Table 3:** Cluster-aware generalized estimating equation results. Primary models included main effects and all two-way interactions; the three-way interaction is an exploratory sensitivity analysis. Wald chi-square tests are reported.

Outcome	Effect	Wald *χ*^2^	df	*p*
Prabhu score	Chelator overall	196.74	14	<0.001
Prabhu score	Activation overall	234.95	20	<0.001
Prabhu score	Root third overall	375.52	14	<0.001
Prabhu score	Chelator×activation	42.19	8	<0.001
Prabhu score	Chelator×root third	29.96	4	<0.001
Prabhu score	Activation×root third	59.25	8	<0.001
Prabhu score	Chelator×activation×root third*	97.62	16	<0.001
Clean-tubule %	Chelator overall	240.10	14	<0.001
Clean-tubule %	Activation overall	42.89	20	0.002
Clean-tubule %	Root third overall	192.52	14	<0.001
Clean-tubule %	Chelator×activation	13.69	8	0.090
Clean-tubule %	Chelator×root third	10.03	4	0.040
Clean-tubule %	Activation×root third	18.16	8	0.020
Clean-tubule %	Chelator×activation×root third*	28.34	16	0.029
Tubule diameter	Chelator overall	276.29	14	<0.001
Tubule diameter	Activation overall	102.62	20	<0.001
Tubule diameter	Root third overall	316.91	14	<0.001
Tubule diameter	Chelator×activation	27.84	8	<0.001
Tubule diameter	Chelator×root third	25.78	4	<0.001
Tubule diameter	Activation×root third	26.24	8	<0.001
Tubule diameter	Chelator×activation×root third*	31.02	16	0.013

* Joint factor-block tests include the factor's main-effect coefficients plus all two-way interactions involving that factor (chelator df = 2 + 8 + 4 = 14; delivery/activation df = 4 + 8 + 8 = 20; root third df = 2 + 4 + 8 = 14); they are not simple main-effect tests.

Dentinal tubule diameter also showed significant chelator x delivery/activation, chelator×root-third, and delivery/activation x root-third interactions (all *p* < 0.001). In exploratory full-factorial sensitivity models, the three-way interaction was significant for Prabhu score (*p* < 0.001), clean-tubule percentage (*p* = 0.029), and tubule diameter (*p* = 0.013). These three-way findings were not decomposed into exhaustive chelator-by-protocol cell contrasts because the model is parameter-rich relative to 14 teeth per factorial cell and such *post hoc* decomposition would generate a large multiplicity burden. They are therefore treated as supportive evidence of context dependence rather than as a basis for cell-level claims. Table 3 summarizes the model-level tests.

### 3.3 Chelator effects by root third

Chelator effects were robust across both smear-layer outcomes. In the coronal third, the model-estimated Prabhu score was 0.70 (95% CI 0.51–0.89) for EDTA, 0.23 (0.09–0.36) for citric acid, and 0.16 (0.07–0.24) for maleic acid. EDTA was less favorable than either organic acid after Holm adjustment (both *p* < 0.001), whereas citric and maleic acids did not differ (*p* = 0.308). The corresponding estimated clean-tubule percentages were 54.7%, 85.5%, and 85.2%, respectively; EDTA again differed from both organic acids (both *p* < 0.001), with no citric-vs.-maleic difference (*p* = 0.928).

The middle third showed the same pattern: EDTA had a higher estimated Prabhu score and a lower clean-tubule percentage than 15% citric acid or 7% maleic acid (all Holm-adjusted *p* < 0.001), while the two organic-acids protocols did not differ significantly. In the apical third, all three tested formaulation separated. Estimated Prabhu scores were 2.80 for 17% EDTA, 0.93 for 15% citric acid, and 1.85 for 7% maleic acid, while estimated clean-tubule percentages were 26.1%, 72.7%, and 54.4%, respectively; all three pairwise contrasts were significant after Holm adjustment (all *p* < 0.001). Thus, under the present experimental conditions, 15% citric acid showed the most favorable apical SEM cleanliness, 7% maleic acid was intermediate, and 17% EDTA was least favorable. Model-estimated values and 95% confidence intervals are shown in Table 4.

**Table 4:** Model-estimated marginal means by chelator and root third. Lower Prabhu scores and higher clean-tubule percentages indicate greater canal-wall cleanliness. Values in parentheses are 95% confidence intervals.

Root third	Chelator	Prabhu EMM (95% CI)	Clean-tubule % (95% CI)	Diameter, µm (95% CI)
Coronal	EDTA	0.70 (0.51–0.89)	54.7 (48.4–60.9)	1.52 (1.38–1.66)
Coronal	Citric acid	0.23 (0.09–0.36)	85.5 (81.7–88.6)	2.71 (2.53–2.89)
Coronal	Maleic acid	0.16 (0.07–0.24)	85.2 (79.4–89.6)	2.82 (2.57–3.07)
Middle	EDTA	1.75 (1.50–2.00)	55.3 (49.6–60.9)	1.50 (1.36–1.63)
Middle	Citric acid	0.95 (0.74–1.17)	84.8 (81.9–87.4)	2.57 (2.40–2.74)
Middle	Maleic acid	0.73 (0.51–0.95)	81.6 (77.9–84.8)	2.69 (2.45–2.93)
Apical	EDTA	2.80 (2.66–2.93)	26.1 (21.2–31.7)	0.78 (0.64–0.91)
Apical	Citric acid	0.93 (0.69–1.17)	72.7 (67.6–77.4)	1.56 (1.39–1.74)
Apical	Maleic acid	1.85 (1.65–2.05)	54.4 (46.9–61.8)	1.23 (1.03–1.43)

### 3.4 Delivery/activation effects and dependence on the assessment method

Delivery/activation protocol effects were strongest for the Prabhu outcome and changed by root third. PUI had the lowest estimated Prabhu score in the coronal (0.02), middle (0.42), and apical (0.83) thirds. In the coronal third, PUI was significantly more favorable than syringe irrigation, manual dynamic activation, EndoActivator, and EndoVac after Holm adjustment; syringe irrigation was also less favorable than the other active/delivery approaches in several contrasts. In the middle third, syringe irrigation was less favorable than all four alternatives, while PUI was more favorable than manual activation and EndoVac and did not differ from EndoActivator. In the apical third, PUI and EndoVac formed the most favorable qualitative group; they did not differ from each other (Holm-adjusted *p* = 0.361), but each was more favorable than manual activation and EndoActivator, and both were substantially more favorable than syringe irrigation.

The quantitative Carvalho method showed a much weaker activation gradient. After Holm correction, the coronal clean-tubule percentage differed only between PUI and syringe irrigation (81.9% vs. 67.0%; adjusted *p* = 0.032), and in the middle third only EndoVac vs. syringe irrigation remained significant (81.7% vs. 68.7%; adjusted *p* = 0.012). No apical quantitative pairwise contrast remained significant after adjustment. These findings explain why the global activation-by-root-third interaction was detectable in the fractional-logit model while the qualitative ranking appeared much stronger. Estimated values for both outcomes, together with tubule diameter, are presented in Table 5 and Figures 2, 3.

**Table 5:** Model-estimated marginal means by irrigant delivery/activation protocol and root third. Lower Prabhu scores and higher clean-tubule percentages indicate greater canal-wall cleanliness. Values in parentheses are 95% confidence intervals.

Root third	Protocol	Prabhu EMM (95% CI)	Clean-tubule % (95% CI)	Diameter, µm (95% CI)
Coronal	Syringe	1.01 (0.79–1.23)	67.0 (58.5–74.6)	2.51 (2.27–2.76)
Coronal	Manual	0.34 (0.18–0.50)	79.8 (72.3–85.7)	2.17 (1.96–2.38)
Coronal	Endo Activator	0.28 (0.15–0.41)	78.7 (72.5–83.9)	2.16 (1.91–2.41)
Coronal	PUI	0.02 (0.00–0.06)	81.9 (75.5–87.0)	2.10 (1.89–2.31)
Coronal	EndoVac	0.59 (0.37–0.81)	78.2 (69.7–84.7)	2.79 (2.46–3.12)
Middle	Syringe	2.10 (1.84–2.36)	68.7 (61.8–74.9)	2.36 (2.13–2.59)
Middle	Manual	1.11 (0.82–1.41)	72.7 (67.4–77.4)	1.89 (1.68–2.11)
Middle	Endo Activator	0.74 (0.49–1.00)	76.4 (71.5–80.7)	2.24 (2.02–2.46)
Middle	PUI	0.42 (0.16–0.68)	78.0 (71.6–83.3)	1.98 (1.80–2.15)
Middle	EndoVac	1.47 (1.17–1.76)	81.7 (76.8–85.8)	2.79 (2.46–3.12)
Apical	Syringe	2.85 (2.69–3.00)	50.7 (42.4–59.1)	1.65 (1.45–1.85)
Apical	Manual	2.23 (1.96–2.49)	51.5 (42.6–60.2)	1.17 (0.92–1.43)
Apical	Endo Activator	2.39 (2.15–2.63)	51.3 (43.1–59.5)	1.01 (0.80–1.22)
Apical	PUI	0.83 (0.58–1.07)	56.7 (46.8–66.0)	1.00 (0.83–1.17)
Apical	EndoVac	1.07 (0.80–1.34)	44.7 (35.4–54.4)	1.12 (0.87–1.37)

**Figure 2:**
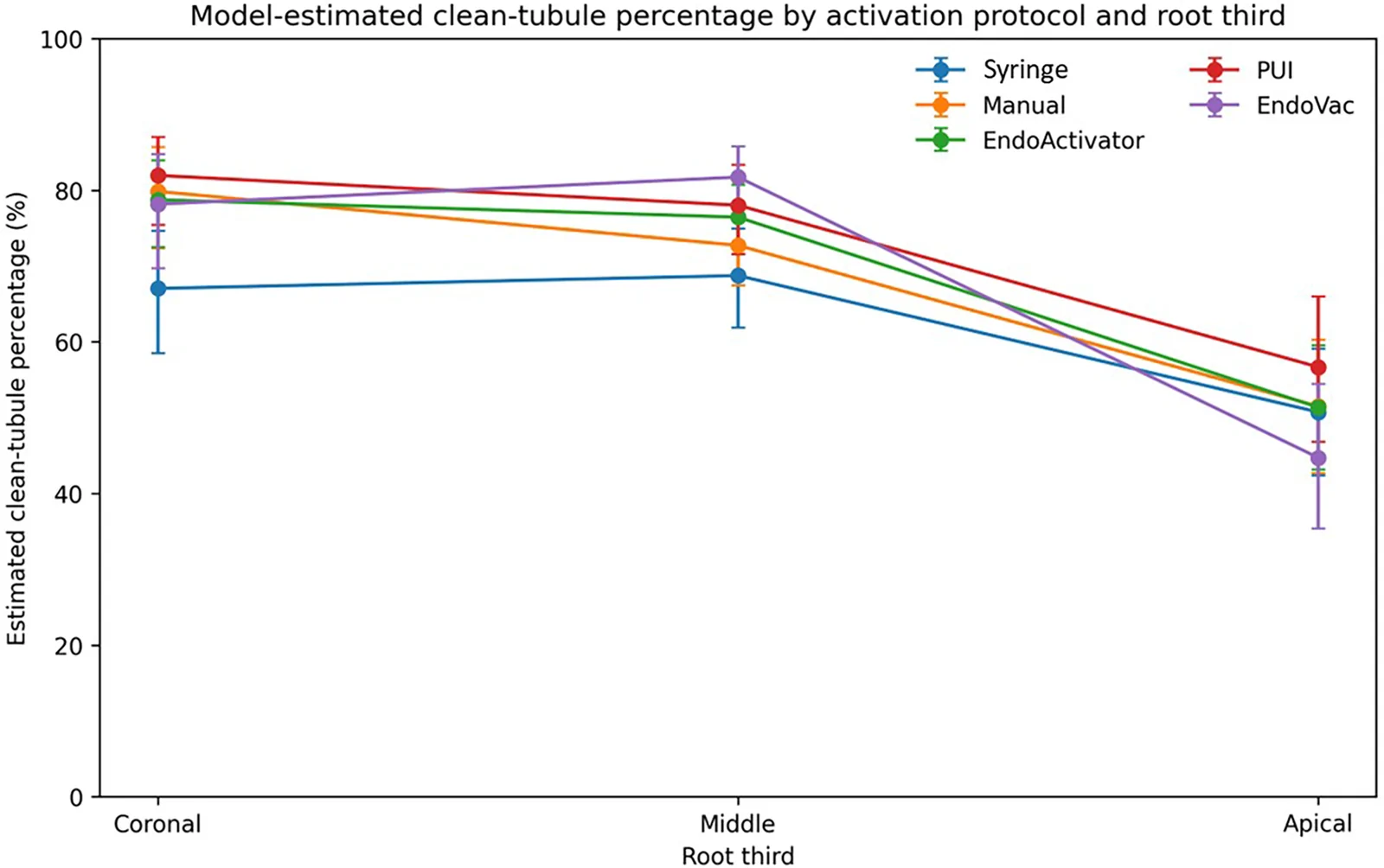
Model-estimated prabhu scores with 95% confidence intervals by root third and delivery/activation protocol. Lower scores indicate less smear layer. Estimates are marginal over chelator levels from the primary ordinal GEE.

**Figure 3:**
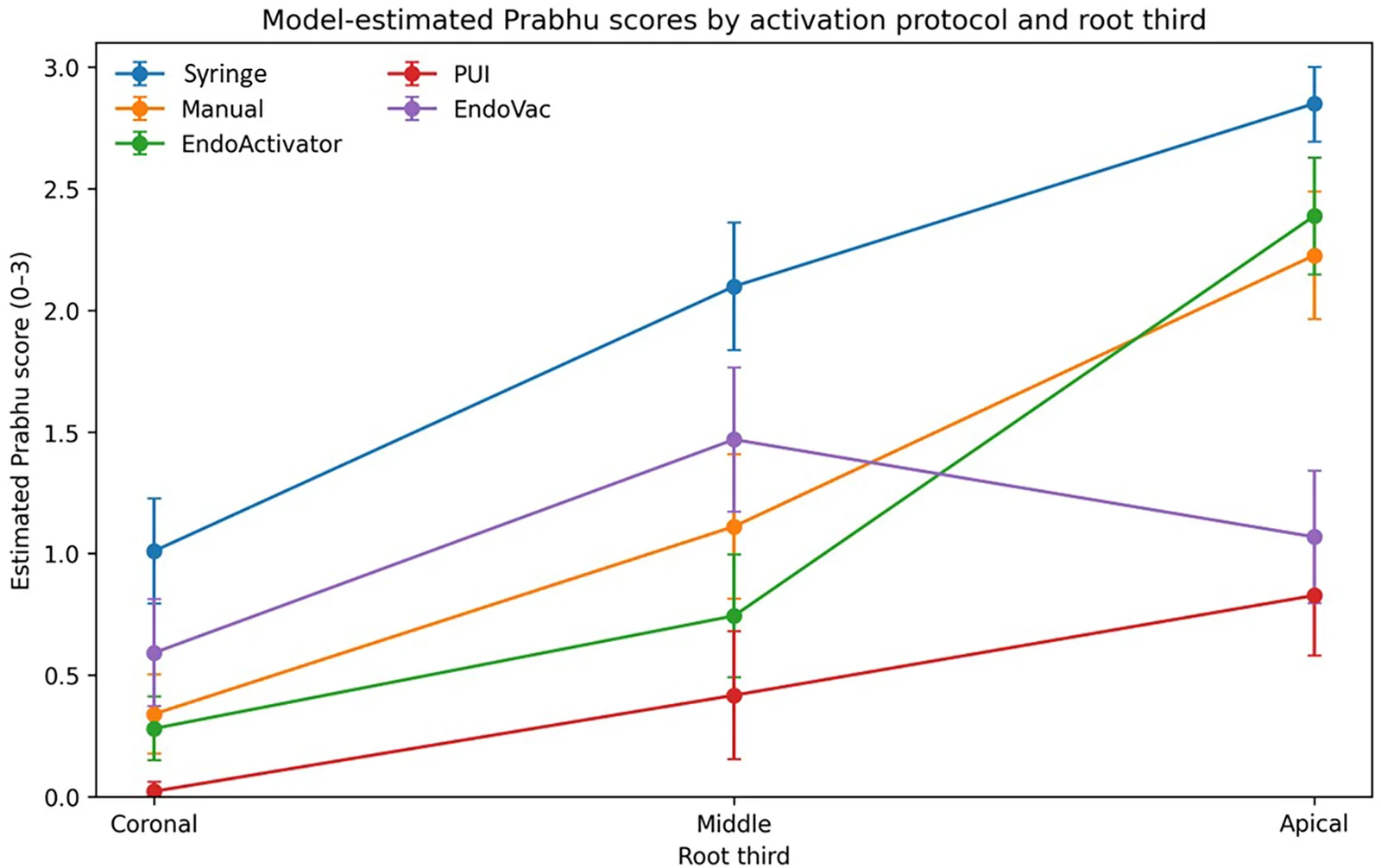
Model-estimated clean-tubule percentages with 95% confidence intervals by root third and delivery/activation protocol. Higher percentages indicate greater canal-wall cleanliness. Estimates are marginal over chelator levels from the fractional-logit GEE.

### 3.5 Dentinal tubule diameter

Mean tubule diameter varied with all three experimental factors and their pairwise interactions. By chelator, EDTA produced smaller model-estimated diameters than citric and maleic acids in the coronal and middle thirds (all Holm-adjusted *p* < 0.001), while citric and maleic acids did not differ in those two thirds. Apically, all three chelators differed: citric acid produced the largest estimated diameter (1.56 µm), followed by maleic acid (1.23 µm) and EDTA (0.78 µm). By activation protocol, EndoVac produced comparatively large coronal and middle diameters, whereas syringe irrigation produced the largest apical estimate (1.65 µm). Because a larger visible lumen may reflect either effective smear removal or loss of peritubular dentin, diameter was treated as an exploratory morphologic outcome rather than a direct measure of cleaning or erosion.

### 3.6 Association and agreement between SEM assessment methods

Across all 630 observations, the finalzed Prabhu value and clean-tubule percentage showed a moderate inverse association (Spearman *ρ* = −0.486, 95% CI −0.547 to −0.420), as expected because higher Prabhu values indicate more smear whereas higher quantitative percentages indicate greater cleanliness. Associations were weaker in the coronal (*ρ* = −0.365) and middle (*ρ* = −0.342) thirds and somewhat stronger apically (*ρ* = −0.451). A secondary weighted-*κ* sensitivity analysis after pragmatic recategorization yielded *κ* = 0.346 (95% CI 0.283–0.400). Because the constructed quantitative thresholds do not naturally correspond to the Prabhu definitions, this *κ* value is reported only as a threshold-dependent sensitivity result and not as evidence that either method is more valid.

### 3.7 Representative SEM findings

Figure 4 shows some significant images of findings obtained using electron microscopy.

**Figure 4:**
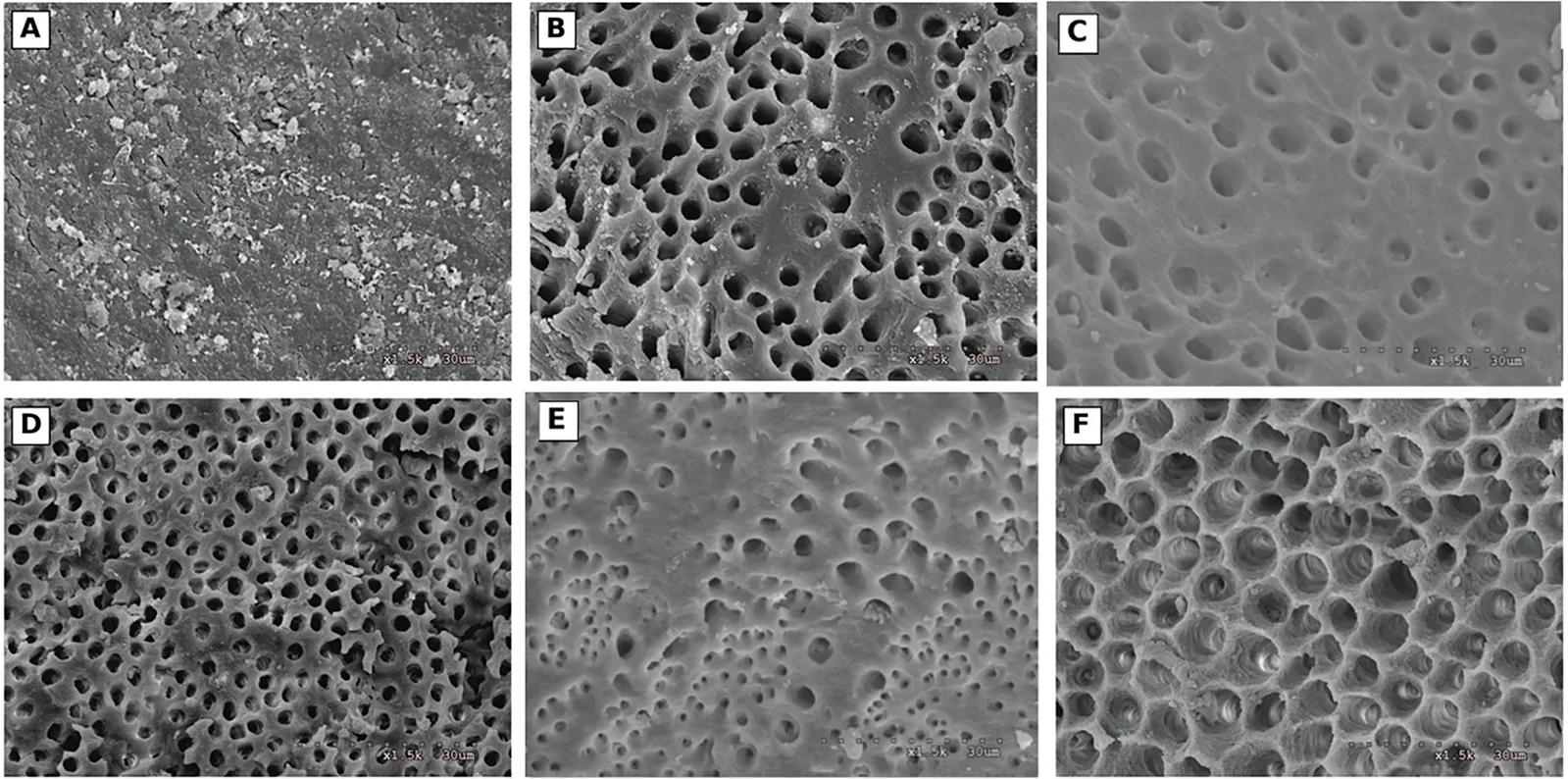
Representative scanning electron micrographs at ×1,500 (scale ba*r* = 30 µm). **(A)** Control–syringe, coronal third; **(B)** 15% citric acid–syringe, coronal third; **(C)** 15% citric acid–manual activation, apical third; **(D)** 15% citric acid–Endo Activator, coronal third; **(E)** 15% citric acid–PUI, apical third; **(F)** 15% citric acid–EndoVac, coronal third.

## 4 Discussion

The principal finding of this randomized factorial *ex vivo* study is that smear-layer removal was context dependent. Once the three root levels from each tooth were modeled as correlated observations, significant interactions emerged among chelator, delivery/activation protocol, and root third. Chelator selection remained the most consistent determinant across both the Prabhu and quantitative clean-tubule outcomes. By contrast, the apparent magnitude of the delivery/activation effect depended strongly on root level and on how SEM images was quantified. This distinction is important because a technique that appears clearly superior on a global visual score may show a much smaller advantage when the outcome is the proportion of patent tubules. This discrepancy is scientifically informative, but it does not establish that one image-analysis method is closer to biological truth than the other.

The chelator results were consistent across the two SEM cleanliness outcomes. In the coronal and middle thirds, 17% EDTA produced less favorable model-estimated cleanliness than 15% citric-acid and 7% maleic-acid protocols, with no meaningful difference between the two organic-acids protocols at those levels. Apically, 15% citric acid under the present experimental conditions was most favorable, 7% maleic acid was intermediate, and 17% EDTA was least favorable by both measurement approaches. Ballal et al. reported superior apical smear removal with 7% maleic acid compared with 17% EDTA (8), and subsequent reviews have continued to identify maleic acid as a promising alternative while emphasizing major variation in concentration, exposure time, irrigant sequence, and SEM scoring (7, 10). Citric-acid activity is likewise concentration- and protocol-dependent activity (6).

The apical advantage of the tested 15% citric-acid protocol over the tested 7% maleic-acid protocol differs from studies favoring maleic acid in the apical third (8, 10). Several protocol-specific mechanisms may contribute, including pH, calcium-complexation kinetics, surface tension, contact time, apical preparation size, replenishment of active solution, and the subsequent NaOCl exposure. The three formulations were tested at established but chemically non-equivalent concentrations, so the present data compare complete clinical/laboratory protocols rather than intrinsic chelating potency. The closed-ended model was intended to reproduce the hydrodynamic difficulty of apical irrigant exchange. Consequently, the finding should be interpreted as a property of the tested protocol rather than evidence that citric acid is universally or clinically superior.

A later independent study from the same research line compared EDTA and HEBP in a separate cohort of 105 teeth using sonic and ultrasonic activation and the Carvalho method (24). That study involved different chelators, fewer delivery/activation systems, and a separate sample; no observations from the present cohort were included. The current study therefore contributes a broader three-chelator by five-protocol factorial comparison. Its clinical implications remain restrained: improved SEM cleanliness may facilitate irrigant access and sealer interaction, but it cannot establish healing, postoperative pain reduction, tooth survival, or superiority of one protocol in patients. Contemporary reviews of endodontic irrigation emphasize that disinfection, fluid dynamics, and dentin-substrate conditioning should be considered together (13). More broadly, systematic evaluation of *in vitro* biological evidence in endodontics shows that laboratory cytotoxicity findings are strongly method-dependent and require cautious clinical contextualization (25). Safety, dentin preservation, irrigant interactions, extrusion risk, and compatibility with obturation must therefore accompany cleanliness when clinical decisions are made.

Beyond smear-layer removal, maleic acid has shown lower *in vitro* cytotoxicity than EDTA at comparable tested concentrations (26), and it has demonstrated antimicrobial activity against Enterococcus faecalis biofilm, both alone and in combination with cetrimide (27). These biological findings do not establish clinical superiority, but they broaden the rationale for evaluating organic acids as alternatives to EDTA. The present factorial design extends these comparisons by showing that the relative effect of a chelator cannot be separated completely from hydrodynamics and canal level.

Anatomical variation may also contribute to root-level differences. Dentinal tubule density and branching vary according to location within the tooth (28), while root-canal dentin shows regional differences in tubule density, orientation, and the surface response to etching (29). The apical region is particularly heterogeneous, with irregular tubule density and direction and even areas devoid of tubules (30). The present specimens were not characterized retrospectively by tooth type, standardized curvature measurement, two-projection radiography, CBCT, or micro-CT; therefore, unmeasured variation in canal cross-sectional geometry, isthmuses, accessory anatomy, dentin thickness, and surface area may have contributed to between-group variability. Random allocation reduces but does not eliminate this possibility in 15 cells of 14 teeth each.

PUI produced the most favorable Prabhu estimates at all three root levels, which is biologically plausible because ultrasonic oscillation promotes acoustic streaming and solution exchange (14–18). However, the quantitative analysis substantially attenuated this apparent advantage. After multiplicity correction, only PUI vs. syringe irrigation remained significant coronally, only EndoVac vs. syringe irrigation remained significant in the middle third, and no apical quantitative activation contrast was significant. The cluster-aware model nevertheless identified a significant delivery/activation-by-root-third interaction. Importantly, the prognostic review by Chubb emphasized that most irrigation studies evaluate *ex vivo* or intermediate endpoints and that high-level evidence linking specific irrigation strategies to root-canal treatment success remains insufficient (31). At the laboratory level, a recent apical SEM comparison of chelating-solution activation methods also found that several active approaches improved smear-layer removal relative to conventional irrigation, without a clear separation among the active devices (32). Both the Prabhu and Carvalho results in the present study arise from the same single selected SEM field per third; therefore, their disagreement reflects different weighting of that sampled field and does not identify a superior reference method. Overall, delivery/activation modifies the spatial pattern of cleanliness, but the apparent effect size depends on root level and image endpoint rather than supporting a single universally superior system.

Tubule diameter provided a complementary but deliberately exploratory morphologic signal and should be elevated as a cautionary counterpoint to the cleanliness findings. The organic-acids protocols were associated with larger visible tubule diameters than 17% EDTA, and in the apical third the model-estimated diameter was 1.56 µm for 15% citric acid vs. 0.78 µm for 17% EDTA. A larger visible lumen may indicate more complete smear removal and exposure of patent tubules, but it may also reflect loss of peritubular mineral and more extensive demineralization. Moreover, the final NaOCl application followed chelation in every experimental protocol and may have contributed to the final surface morphology. Contemporary evidence shows that EDTA, citric acid, maleic acid, and other chelators can alter microhardness and mineral content depending on concentration and exposure (5, 11–13). The diameter measurement is additionally susceptible to measurement-selection bias because heavily smeared fields expose fewer measurable tubules. Accordingly, the protocol that appears most favorable for smear removal cannot be considered ‘optimal’ on the basis of the present data. Direct erosion scoring, EDS, nano hardness testing, or three-dimensional surface methods would be required to distinguish desirable tubule exposure from excessive substrate alteration. Future studies should therefore complement tubule-diameter measurements with direct substrate-preservation outcomes, including microhardness testing, energy-dispersive x-ray spectroscopy (EDS) of mineral composition, and/or validated direct erosion grading scales. Until such complementary assessments are available, wider tubule orifices should not be interpreted as an optimal clinical endpoint.

The direct comparison between image-assessment methods is a methodological contribution of the study. The moderate inverse Spearman association confirms that the finalized Prabhu value and continuous clean-tubule percentage capture related aspects of the same sampled surface condition. The weighted-*κ* analysis is less definitive because the quantitative categories were constructed pragmatically and do not map naturally onto the Prabhu thresholds; it should therefore be regarded only as a sensitivity analysis. The 0%–40% quantitative category resulted from pooling the original Carvalho “dirty” and “very dirty” bands only to reduce the five-level Carvalho descriptive scheme to four ordered levels; this pooling does not imply biological equivalence with Prabhu score 3. Previous work comparing expert scoring with digital image analysis has reported different levels of agreement under other scoring and image-processing conditions (33). In addition, both methods here were applied to one selected ×1,500 field per root third, so disagreement cannot establish which method is more valid. Global ordinal scoring and restricted-window tubule counting simply weight visual information differently. Future SEM studies should prespecify a primary image endpoint, use coordinate-based or otherwise reproducible field selection, acquire multiple fields per region where feasible, report observer calibration and reliability, and consider reproducible semi-automated segmentation.

This study presents several limitations. First, the *ex vivo* model cannot replicate clinical conditions such as biofilm maturity, temperature, tissue buffering capacity, pulp/periapical tissues, host response, or dynamic fluid conditions. Second, factors such as tooth type distribution, standardized root curvature measurements, canal cross-sectional geometry, dentin thickness, canal surface area, and preoperative radiographic/CBCT/micro-CT standardization were not taken into account; therefore, anatomical heterogeneity may persist despite randomization. Third, scanning electron microscopy (SEM) is a destructive technique, and samples rely on selected two-dimensional fields. Only one field at 1,500× magnification was analyzed per root third, and the exact criteria for field selection within each third were not documented; this introduces potential field-selection bias and precludes inferences regarding the cleanliness of the entire canal. Fourth, although two independent, blinded observers initially evaluated the images and their average was used, the individual ratings from each observer are no longer available, making it impossible to recalculate inter-observer reliability. Fifth, the original CDT/DDT counts and the formal criteria for borderline tubules were not preserved; consequently, the quantitative outcome variable was modeled as a fractional percentage rather than binomial counts, and it is not possible to audit specific details regarding counting reproducibility. Sixth, tubule diameter measurements were limited to visible tubules—introducing potential measurement selection bias—and no direct assessment of erosion, mineral loss, microhardness, or three-dimensional surface characteristics was performed. Consequently, it is not possible to distinguish between larger diameters representing desirable exposure and those resulting from excessive demineralization. Seventh, the three chelators were tested at specific concentrations using a sequence that included NaOCl after chelation; thus, the results apply to the tested protocols rather than to exposures involving chemically equivalent chelators. Finally, significant three-way interactions were observed in exploratory sensitivity models—with 14 teeth per factorial cell—but were not broken down into exhaustive cell-level comparisons due to limited precision and the issue of multiplicity. These limitations are offset by a comparatively large total sample, balanced randomized factorial allocation, paired assessment of three root levels per tooth, dual SEM assessment strategies, multiplicity-adjusted contrasts, and modeling that accounts for data clustering (intra-tooth dependence).

## 5 Conclusions

Within the limitations of this randomized factorial *ex vivo* SEM study, chelator selection had a consistent effect on smear-layer removal, whereas the effect of irrigant delivery/activation depended on root third and image-assessment method. The 15% citric- acid and 7% maleic-acid protocols were more favorable than 17% EDTA in the coronal and middle thirds, and under the present experimental conditions 15% citric acid showed the most favorable apical SEM cleanliness. PUI produced the lowest Prabhu values, but its advantage was less consistent when cleanliness was quantified as the percentage of patent tubules, with no significant apical quantitative pairwise delivery/activation contrast after multiplicity adjustment. Larger tubule diameters with the organic-acid protocols may represent more complete tubule exposure, greater dentin alteration, or both; because erosion was not directly measured, the study cannot identify an optimal chelator. The two SEM assessment approaches were associated but should not be treated as interchangeable or ranked for validity from these data. These findings support protocol-specific interpretation and future studies that standardize anatomy and SEM field selection, model repeated observations within teeth, and combine cleanliness with direct measures of dentin preservation. They do not establish clinical superiority of any irrigant or activation protocol.

## Data Availability

The raw data supporting the conclusions of this article will be made available by the authors, without undue reservation.
